# Bactericidal Activity of an Imidazo[1, 2-*a*]pyridine Using a Mouse *M. tuberculosis* Infection Model

**DOI:** 10.1371/journal.pone.0087483

**Published:** 2014-01-31

**Authors:** Yong Cheng, Garrett C. Moraski, Jeffrey Cramer, Marvin J. Miller, Jeffrey S. Schorey

**Affiliations:** 1 Department of Biological Sciences, Center for Rare and Neglected Diseases and Eck Institute for Global Health, University of Notre Dame, Notre Dame, Indiana, United States of America; 2 Department of Chemistry and Biochemistry, University of Notre Dame, Notre Dame, Indiana, United States of America; 3 Lilly Research Laboratories, Eli Lilly and Company, Indianapolis, Indiana, United States of America; Colorado State University, United States of America

## Abstract

*Tuberculosis* remains a global threat due in part to the long treatment regimen and the increased prevalence of drug resistant *M. tuberculosis* strains. Therefore, new drug regimens are urgently required to combat this deadly disease. We previously synthesized and evaluated a series of new anti-tuberculosis compounds which belong to the family of imidazo[1,2-*a*]pyridines. This family of compounds showed low nM MIC (minimal inhibitory concentration) values against *M. tuberculosis in vitro*. In this study, a derivative of imidazo[1,2-*a*]pyridines, (*N*-(4-(4-chlorophenoxy)benzyl)-2,7-dimethylimidazo[1,2-*a*]pyridine-3-carboxamide) (ND-09759), was selected as a promising lead compound to determine its protective efficacy using a mouse infection model. Pharmacokinetic analysis of ND-09759 determined that at a dosage of 30 mg/kg mouse body weight (PO) gave a maximum serum drug concentration (C_max_) of 2.9 µg/ml and a half-life of 20.1 h. *M. tuberculosis* burden in the lungs and spleens was significantly decreased in mice treated once daily 6 days per week for 4-weeks with ND-09759 compared to untreated mice and this antibiotic activity was equivalent to isoniazid (INH) and rifampicin (RMP), two first-line anti-TB drugs. We observed slightly higher efficacy when using a combination of ND-09759 with either INH or RMP. Finally, the histopathological analysis revealed that infected mice treated with ND-09759 had significantly reduced inflammation relative to untreated mice. In conclusion, our findings indicate ND-09759 might be a potent candidate for the treatment of active TB in combination with current standard anti-TB drugs.

## Introduction

Tuberculosis (TB) caused by intracellular pathogen *M. tuberculosis* (*M.tb*) remains one of the most prevalent and deadly infectious diseases. About one-third of the world’s population are infected with *M. tuberculosis*, and 5 to 10% of infected individuals will develop active TB disease in their lifetime, resulting in approximately 9 million new cases of active disease and 1.5 million deaths per year [1]. Although the global TB incidence is in gradual decline, the control of TB has been hampered by the emergence of multidrug-resistant (MDR) and extensively drug-resistant (XDR) *M. tuberculosis* strains [2]. Patients infected with MDR *M. tuberculosis* need to receive a prolonged therapy consisting of first-line and second-line drugs for at least two years. In practice, long-term treatment is frequently aborted due to toxicity and low tolerability derived from drug-drug interaction or other side effects [3]. Since rifampicin (RMP) was approved approximately 40 years ago, no new antibiotic has been introduced as a first-line drug for treatment of TB [4]. The Stop TB Partnership set a long-term target of eliminating TB as a public health concern by reducing global incidence to less than one case per million by 2050 [5]. It is clear that to achieve this level of TB control, new, effective and safe anti-TB drugs and combination regimens are needed to not only address MDR and XDR but also to shorten treatment duration.

**Figure 1:**
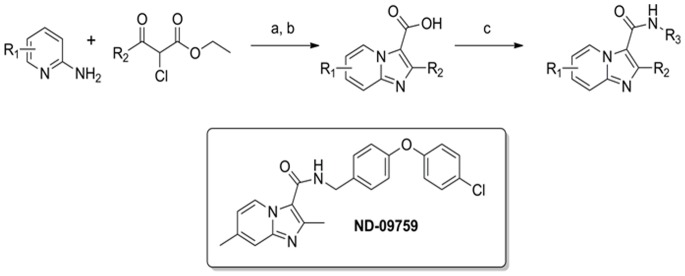
General three step synthesis of IPAs and the chemical structure of ND-09759, *N*-(4-(4-chlorophenoxy)benzyl)-2,7-dimethylimidazo[1,2-*a*]pyridine-3-carboxamide. Reagents: (a) 1,2-Dimethoxyethane, reflux, 48 h.; (b) 1. LiOH, Ethanol, 2. HCl, 56 h.; (c) EDCI, DMAP, (4-(4-chlorophenoxy)phenyl)methanamine, CH_3_CN, 16 h.

One class of compounds that have garnered recent interest as TB antibiotics are the Imidazo[1,2-a]pyridine carboxamides (IPAs). These compounds are readily synthesized by the general scheme shown in figure 1, and have been found to be ideally suited to structure-activity-relationship (SAR) studies with MIC values ranging from low micromolar to low nanomolar levels that are not affected by serum. They are low molecular weight and have ideal log P values (typically <3), good metabolic stability and tunable pharmacokinetics (PK). Moreover this class of compounds seems to be remarkably selective since while they are potently active against *M. tuberculosis*, *M. avium*, and *M. bovis*, they are not active against other mycobacteria, other gram positive or gram negative bacteria. Interestingly, some of the IPAs showed activities comparable to the first-line anti-TB drug RMP but more potent than INH, indicating a potential contribution to novel combination regimens [6]–[8]. The IPAs were also effective against multi-drug resistant strains of *M. tuberculosis* and showed a large therapeutic window assessed through a HepG2 toxicity assay [7]. In a study by Abrahams et al. they also identified and tested four imidazo[1,2-*a*]pyridine compounds which showed good MIC against various strains of *M. tuberculosis* and low toxicity against the HepG2 cell line [9]. Similar low MICs against drug-susceptible and drug-resistant strains were observed with Q203, another IPA [10]. In the current study, we determined the pharmacokinetics and *in vivo* activity of *N*-(4-(4-chlorophenoxy)benzyl)-2,7-dimethylimidazo[1,2-*a*]pyridine-3-carboxamide (ND-09759), (Figure 1) alone or in combination with either isoniazid (INH) or RMP. We chose this particular imidazo[1,2-*a*]pyridine as it showed a low MIC against various strains of *M. tuberculosis* including MDR strains and favorable PK. The results indicate that this drug is efficacious against *M. tuberculosis* in a mouse aerosol infection model with an activity comparable to INH and RMP.

## Materials and Methods

### Animal and Ethic Statement

8-week-old female Balb/c (H-2^d^) mice were purchased from Charles River (MA, USA) and housed at the institutional animal facility under specific-pathogen-free conditions during the experiment. *M. tuberculosis* infection was carried out in the biosafety level 3 laboratory. This study was carried out in compliance with the recommendations in the Guide for the Care and Use of Laboratory Animals of the National Institutes of Health. All animal procedures were approved by the Institutional Animal Care and Use Committee at the University of Notre Dame (Animal Welfare Assurance number A3093-01) and procedures were used to minimize animal pain and suffering. Mice were observed daily for any adverse effects as indicated below.

### Bacteria

Wild type *M. tuberculosis* H37Rv strain was grown in Middlebrook 7H9 broth medium (Difco, Becton-Dickinson) supplemented with 10% OADC (oleic acid/albumin/dextrose/catalase), 0.2% glycerol and 0.05% Tween 80 until exponential phase and then aliquoted and stored at −80 ^o^C until use.

### Anti-TB Compounds

Isoniazid and rifampicin were purchased from Sigma-Aldrich, Co. LLC., MO, USA. ND-09759 was synthesized and purified as described previously (7). All compounds were formulated for oral administration in 80% aqueous propylene glycol prior to treatment.

### Evaluation of Maximum Tolerated Dose

Wild type healthy Balb/c mice (8-week old, female) were randomly divided into 4 groups (n = 5) and then received 24 doses of ND-09759 formulated in 80% aqueous propylene glycol with one dose daily by oral gavage for a period of 4 weeks. The control group was given only 80% aqueous propylene glycol and the remaining groups were injected with ND-09759 at 10 mg/kg, 30 mg/kg or 60 mg/kg body weight. The body weight, clinical signs of distress and mortality of mice were observed daily during the 4-week study. Mice were humanely euthanized by cervical dislocation under anesthesia with isoflurane when they displayed signs of compound-related morbidity such as excessive weight loss, lethargy, severe unrelieved distress, nonabulatory and hypothermia. At the end of study, all mice were humanely euthanized by cervical dislocation under anesthesia with isoflurane.

### Pharmacokinetics of ND-09759 in Mice

The single-dose pharmacokinetics of ND-09759 was determined in uninfected 8-week-old male Balb/c mice. The mice received a single dose of ND-09759 at 30 mg/kg by oral gavage. Three of the mice were anesthetized with isoflurane and cheek blood samples were collected at 0.25, 0.5, 1, 2, 4 and 12 h after initiation of ND-09759 administration. Samples were analyzed by LC-MS.

### Assessment of Compound Efficacy in Mice

Eight-week-old female Balb/c mice were infected with 50–150 CFU (colony forming unit) of *M. tuberculosis* H37Rv by using an Inhalation Exposure System (Glas-Col, Terre haute, IN, USA). Five mice from each batch of *M. tuberculosis* infection were humanely sacrificed by cervical dislocation under anesthesia with isoflurane 1 day after infection and on the day of treatment initiation to determine the level of mycobacteria implanted in the lungs and spleens by aerosol challenge and mycobacterial burden at the starting point of drug treatment, respectively.

The drug treatment was initiated 4 weeks post infection and administered by oral gavage 6 times per week (once per day) for a period of 4 weeks. The drug doses were designed as follow: ND-09759 (30 mg/kg), INH (25 mg/kg) and RMP (20 mg/kg). In the negative control groups, mice were only injected by oral gavage with 80% aqueous propylene glycol.

All mice were sacrificed by cervical dislocation under anesthesia with isoflurane 1 day after the final dosing and the lung and spleen homogenate was prepared in phosphate-buffered saline (PBS) containing 0.05% (vol/vol) Tween 80. The tissue homogenate was appropriately diluted in the same buffer, and then 50 µl of the diluted homogenate was spread on Middlebrook 7H11 agar plates with 10% OADC, 0.5% glycerol and 0.05% Tween 80, and containing a cocktail of fungizone and PANTA (polymixin B, amphotericin B, nalidixic acid, trimethoprim, and azlocillin). *M. tuberculosis* colonies were counted after 3–4 weeks of incubation at 37 ^o^C and expressed as log_10_ CFU per organ.

### Histopathology

The lung samples were first harvested and fixed in 10% neutral buffered formalin (Fisher Scientific, Fair Larn, NJ) at room temperature, and then embedded in paraffin. The sections at 5 µm thickness were stained with hematoxylin and eosin or processed with acid fast staining. To evaluate the lung inflammation and damage, the histopathology sections were scored for severity by scanning multiple random fields in 3 sections of each tissue per mouse based on the extent of granulomatous inflammation as described [11]: 0 = no lesion, 1 = minimal lesion (1–10% area of tissue in section involved), 2 = mild lesion (11–30% area involved), 3 = moderate lesion (31–50% area involved), 4 = marked lesion (50–80% area involved), 5 = severe lesion (>80% area involved).

### Statistical Methods

The data obtained was analyzed by ANOVA. Differences between means were assessed for significance by Tukey’s test. A value of p≤0.05 was considered significant. The computer program GraphPad PRISM 5 was used for these tests.

## Results

### Maximum Tolerated Dose (MTD) of ND-09759 in Mice

**Figure 2:**
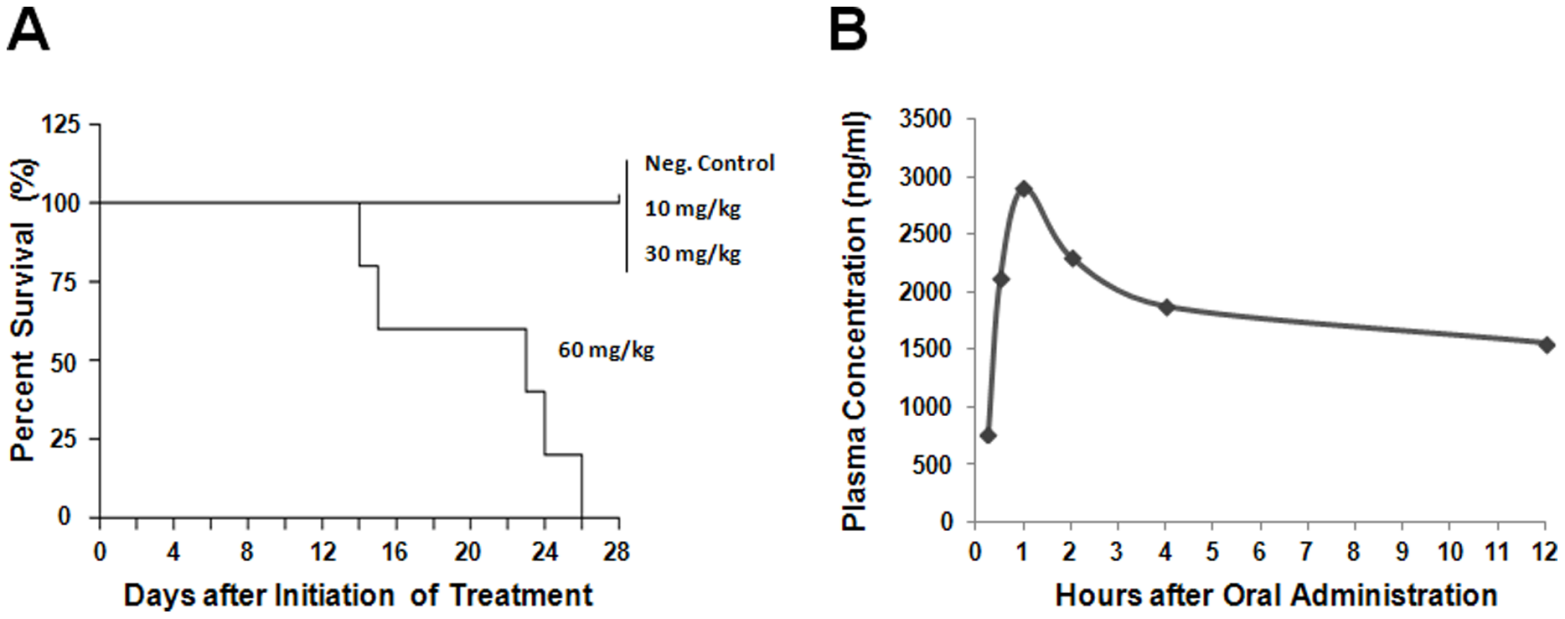
Maximum Tolerated Dose and Pharmacokinetics of ND-09759 in mice. (A) Mouse survival curves from maximum tolerated dose study. All mice (n = 5) received one dose daily 6 times weekly for 4 weeks. 80% aqueous propylene glycol, (vehicle control). (B) Mean serum concentration of ND-09759 at different times post-treatment (±standard deviations) in Balb/c mice (n = 5) after a single oral dose (30 mg/kg).

A maximum tolerated dose test was performed to determine the amount of ND-09759 which could be administered. Mice were administrated with the compound by oral gavage once daily at concentrations of 10 mg/kg, 30 mg/kg, or 60 mg/kg body weight 6 times per week over a 4 week period. The mice were observed daily for body weight, behavioral changes, clinical signs of distress and mortality on the basis of the criteria recommended by Laboratory Animal Science Association (LASA). The mice tolerated a dose up to 30 mg/kg with no significant changes in animal behavior and no clinical signs of distress (data not shown). In contrast, the mice subjected to a highest dose of ND-09759 (60 mg/kg) showed signs of clinical distress including loss of body weight (data not shown). All mice at the 60 mg/kg dose died or were euthanized by the end of the toxicity study (Fig. 2A). Therefore, the MTD study for ND-09759 indicated that the maximum tolerated dose was between 30 and 60 mg/kg in wild type Balb/c mice. We used a 30 mg/kg/day dose for the *in vivo* efficacy assays.

### Single-dose Pharmacokinetics of ND-09759 in Mice

Half-life and sequential exposure time of drug in serum is critical for its *in vivo* efficacious activity. Oral administration of ND-09759 at a dose of 30 mg/kg in Balb/c mice produced a maximum serum concentration (C_max_) of 2.9 µg/ml at 1 h after compound administration, resulted in an AUC_0–24_ of 22.2 µg h/ml during the entire PK study, and showed a half-life of 20.1 h, displaying a marked bioavailability (Fig. 2B).

### Protective Efficacy of ND-09759 against TB in a Mouse *M. tuberculosis* Infection Model

**Figure 3:**
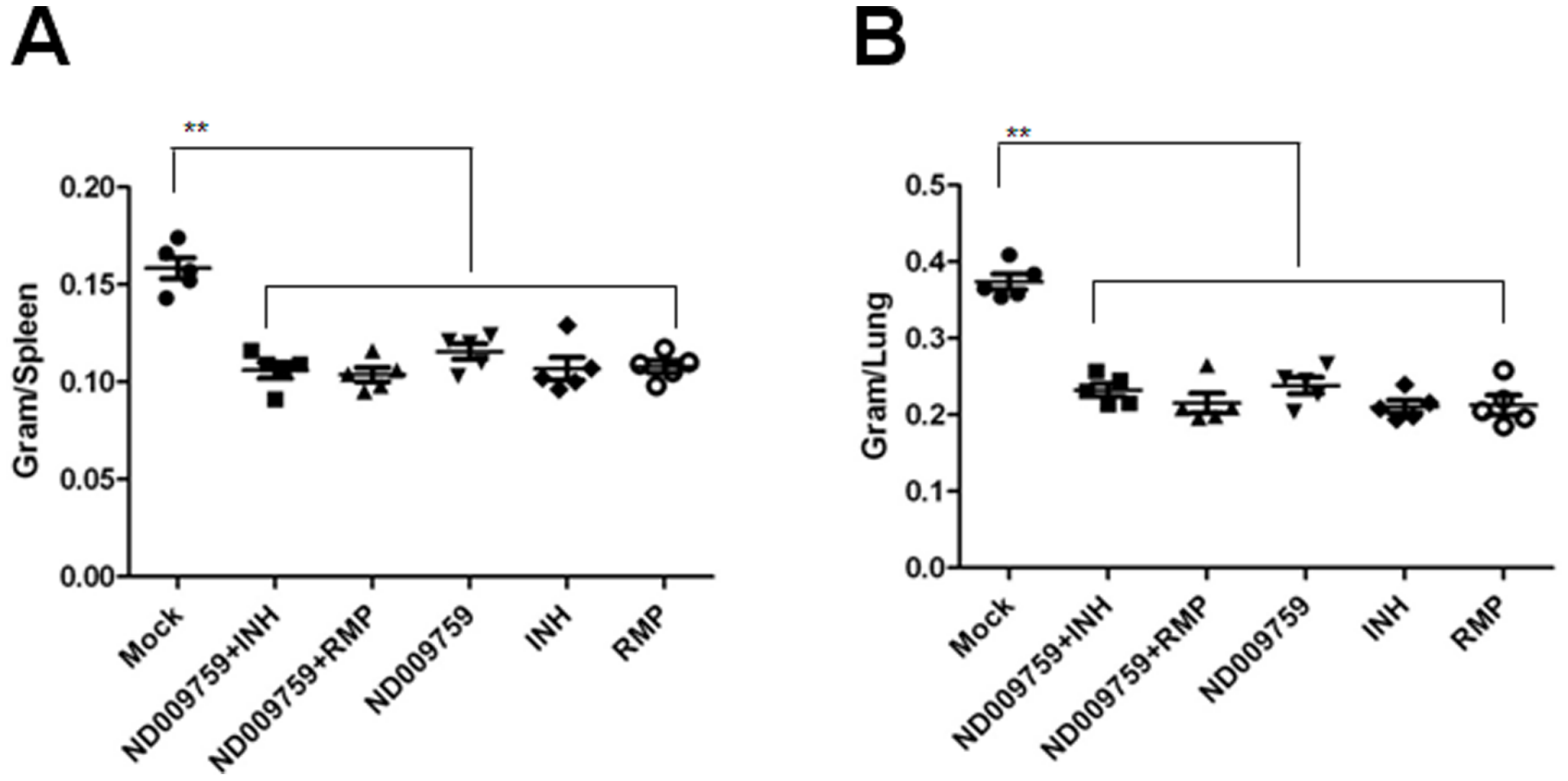
Lung (A) and spleen (B) weight of *M. tuberculosis*-infected Balb/c mice (n = 5) treated with ND-09759 alone or in combination with INH or RMP. Mock, 80% aqueous propylene glycol, (vehicle control), Bars represents means ±standard deviation. **, P<0.05 compared to no treatment group.

**Figure 4:**
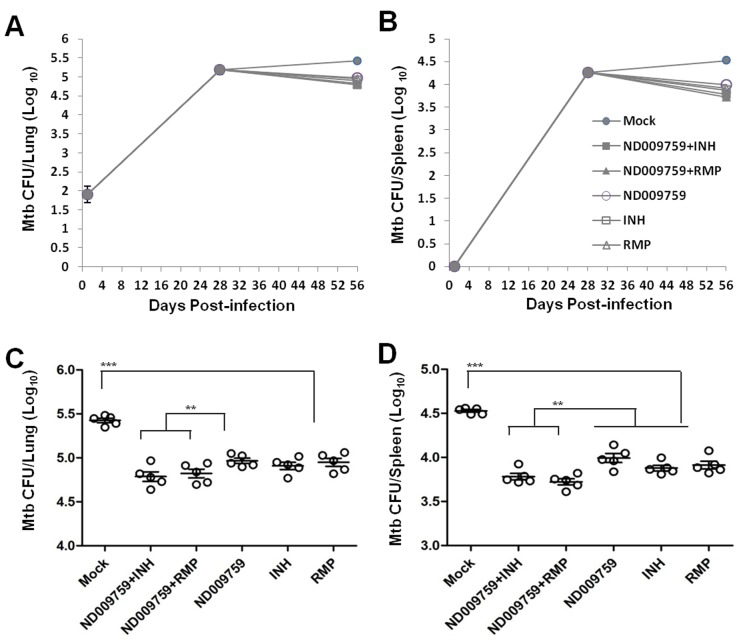
Mycobacterial counts in the lungs and spleens of *M. tuberculosis*-infected Balb/c mice after treatment with ND-09759 or combination regimens. Balb/c mice (n = 5) were infected with *M. tuberculosis* H37Rv by aerosol challenge and then treated by oral gavage with 80% aqueous propylene glycol (solid cycle, vehicle control), ND-09759 (cycle), ND-09759 plus INH (solid square), ND-09759 plus RMP (solid triangle), INH (square) and RMP (triangle), respectively, as described in *Materials and Methods*. The results show the log_10_ CFU in the lung (A and C) and spleen (B and D). C and D, Mycobacterial counts after the completion of chemotherapy and each open cycle represents an individual mouse. ***, p<0.05 compared to no treatment group; **, P<0.05 between ND-09759 alone and ND-09759+ INH or RMP.

To evaluate the efficacies of ND-09759 alone or in combination with INH or RMP we used a low-dose aerosol *M. tuberculosis* infection in a Balb/c mouse background. One day after the aerosol infection, a group of 5 mice were sacrificed and the initial bacterial load was determined. The mean bacterial concentration one day post-infection was 79+/−22 in the lung while no bacteria were detected in the spleen 1 day post-infection. At the start of chemotherapy (4 weeks post-infection) another group of 5 mice was sacrificed and the bacterial load in the lung and spleen determined. During the course of chemotherapy, no mortality related to *M. tuberculosis* infection or drug reaction occurred and the mice did not show overt signs of stress. As shown in figure 3, upon completion of therapy, all compound-treated mice showed spleens and lungs of diminished weight/size compared to organs isolated from infected but untreated mice. Moreover, all mice receiving ND-09759 alone or combination regimens had significantly reduced *M. tuberculosis* burden in both lungs and spleens relative to negative control mice (Fig. 4A–D). Interestingly, the *M. tuberculosis* counts in the lungs and spleens of mice treated with ND-09759 alone were comparable to those in the groups receiving either INH or RMP alone. Furthermore, the *M. tuberculosis*-infected mice treated with ND-09759 plus INH or RMP exhibited a small but significant decrease in the *M. tuberculosis* CFU in both lungs and spleens compared to those treated with ND-09759 alone, indicating an enhanced drug activity for the combined regimens (Fig. 4C–D). Since the *M. tuberculosis* counts at the initiation of chemotherapy were 5.18 log_10_ in the lungs and 4.26 log_10_ in the spleens, ND-09759 significantly reduced *M. tuberculosis* bacterial load by approximately 0.27 log_10_ in the spleen and 0.22 log_10_ in the lung, demonstrating a bactericidal activity in mice. This activity was comparable to that of INH and RMP which reduced *M. tuberculosis* bacterial load by 0.38 (INH) and 0.35 (RMP) log_10_ in the spleen and 0.27 (INH) and 0.23 (RMP) log_10_ in the lung.

**Figure 5:**
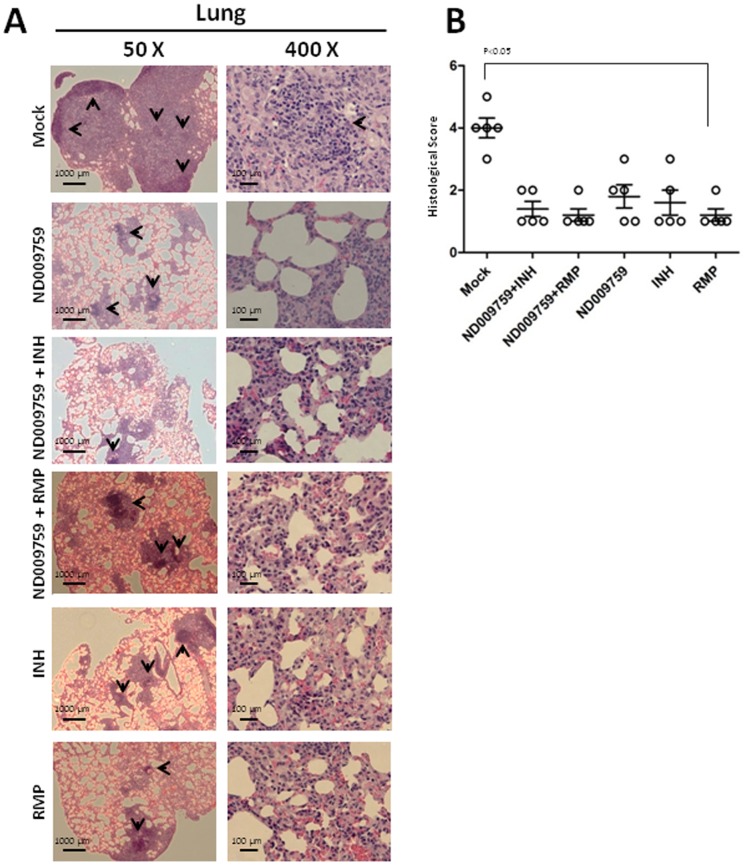
Lung histopathology in drug-treated and untreated infected mice. (A) Images are shown for TB-infected Balb/c mice treated with 80% aqueous propylene glycol (mock), ND-09759 alone, ND-09759 plus INH or RMP, or with INH or RMP alone. The left and right panels show low power (original magnification 50X) and high power (original magnification 400X) of H&E-stained lung sections, respectively. Sections are representative of lung sections from 5 mice per group and from one of two independent experiments. (B) Quantitative scoring of histopathology. Bars represent means ±standard deviation.

**Figure 6:**
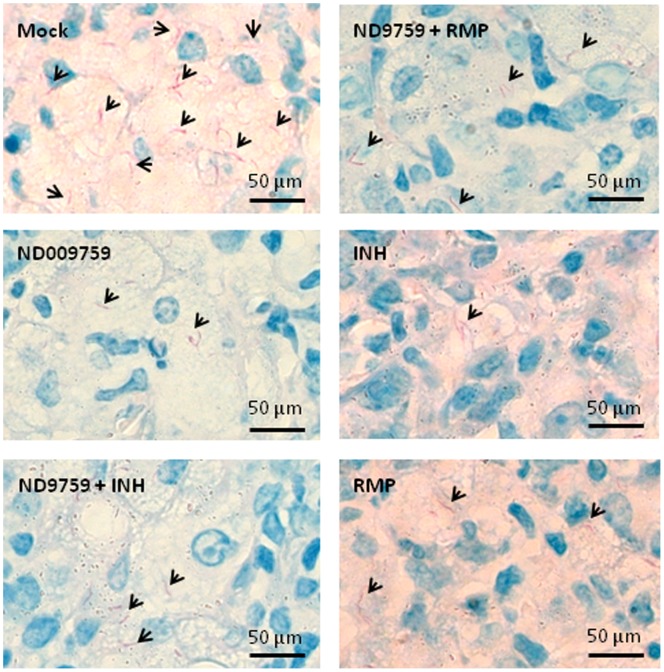
Acid-fast staining of lung sections from *M. tuberculosis*-infected Balb/c mice (n = 5) following treatment with 80% aqueous propylene glycol (vehicle control) or chemotherapy with drugs as indicated. Magnification (630X). Acid fast bacilli indicated by arrows.

*M. tuberculosis* infection induces continued lung inflammation resulting in significant pathology over time [12]. Therefore, pathological changes in mice were evaluated by sectioning lung tissue and staining with hematoxylin and eosin. Histological analysis of the *M. tuberculosis*-infected mouse lungs showed untreated mice had seriously compact inflammatory lesions characterized by mononuclear infiltration (Fig. 5A). In contrast, the mice treated with ND-09759 alone or in combination with either INH or RMP showed limited granulomatous inflammation and their pulmonary lesions were discrete and surrounded by largely normal lung tissue with minimal interstitial pneumonia (Fig. 5A). No obvious histological difference between the various drug treatment regimens was observed. A further quantitative analysis based on a visual scoring system [11] demonstrated that all compound-treated mice showed significantly reduced histological score compared to untreated mice which had moderate-to-severe diffuse granulomatous pneumonia in the lungs (Fig. 5B). Finally, consistent with the CFU counts, the numbers of acid fast stained bacilli detected in lung sections from compound-treated mice were minimal compared to the infected but untreated mice (Fig. 6).

## Discussion

ND-09759 belongs to a novel class of drug compounds, imidazo[1,2-*a*]pyridines, that exhibit potent activity against *M. tuberculosis*. Its *in vivo* activity combined with its synthetically tractability and druggable properties suggests that this family of compounds could be developed into a new class of anti-TB drugs. More specifically, ND-09759 exhibits a potent MIC activity (MIC ≤0.006 µM or 0.0024 µg/ml) against *M. tuberculosis* significantly lower than the reference anti-TB drugs INH (0.3 µM), comparable to RMP (0.003 µM) [7], and similar to or better than other anti-TB compounds including TMC 207 (0.030−0.120 µg/ml) and Delamanid (0.006–0.024 µg/ml) [13], SQ109 (0.7–1.56 µM) [14], PA-824 (0.015–0.25 µg/ml) [15], and Linezolid (0.25–1 µg/ml) [16], which are currently under phase II or III clinical trial [17]. In addition, pharmacokinetic analysis showed that ND-09759 has a good oral bioavailability in mice and its PK value is comparable to the standard drugs INH and RMP [18], [19]. When given as a monotherapy, oral administration of ND-09759 at 30 mg/kg once daily for 6 days per week for 4 weeks resulted in 0.5 log_10_ reduction in mycobacterial burden, which was comparable to those of INH at 25 mg/kg and RMP at 20 mg/kg. Third, treatment with ND-09759 at 30 mg/kg in conjugation with either INH at 25 mg/kg or RMP at 20 mg/kg resulted in a small but significant decrease in mycobacterial burden compared to ND-09759 alone suggesting some additive effect. Finally, consistent with CFU counts, oral administration of ND-09759 alone or in combination with either INH or RMP results in a significant decrease in lung pathology compared to infected but untreated mice.

Previously published reports using IPAs found a 2 log decrease in *M. tuberculosis* burden in the lung following an 8 day treatment regimen when compared to infected/untreated control mice, which was a significantly more pronounced reduction than we observed [9]. However in this study they evaluated the IPAs during an acute infection (i.e. started treatment one day after infection) when bacterial numbers were rapidly increasing. Although, this acute model shows that the IPAs can limit bacterial growth, it did not mimic the scenario in which TB antibiotics are used clinically. Moreover, the IPAs tested were bacterostatic as the bacterial burden was slightly higher than the inoculum after the 8 day treatment regimen. In contrast, as indicated above, ND-09759 had bactericidal activity and was equally effective compared to our positive controls INH and RMP. More recent studies by Pethe *et al*. addressed the potency of IPAs against *M. tuberculosis* during an acute and chronic infection model and found their lead compound to limit bacterial growth to a level comparable to Bedaquiline and Isoniazid [9] However, in this study they did not evaluate their IPA in combination with other first line TB drugs.

Our current study assessed the efficacy of ND-09759 against a chronic infection in mice. This is based on the observation that mycobacterial burden increased only 0.3 log_10_ over the 4-week drug evaluation period compared to the 3 log increase during the first 4 weeks of infection. According to the WHO guideline, standard anti-TB combination consists of compounds that have early bactericidal activity (INH), sterilizing activity (RMP and PZA), and an antibiotic to prevent emergence of drug resistant strains (EMB). However, an apparent antagonism between INH and PZA has been described in mouse models but it remains unknown if this holds true in humans. In mice, the removal of INH from the combination provides more effective protection in comparison to the complete regiment, implying an effective substitute of INH might provide benefit to the next generation of standard treatment [20]–[22]. In view of ND-09759’s bactericidal activity, it might provide a complement to INH.

There is a general agreement that the long treatment duration for anti-TB drug regimens contribute to the development of drug-resistant *M. tuberculosis* strains, causing the failure of chemotherapy and continuous *M. tuberculosis* transmission. Any new anti-TB drugs or cocktail regimens that are capable of shortening treatment length have a potential to reduce *M. tuberculosis* transmission and emergence of drug-resistant strains [23]. Based on the aim of global STOP TB Alliance, a new anti-TB drug when combined with existing TB drugs or other new drugs is expected to either shorten treatment duration, improve the therapy of multi-drug/extreme-drug resistant TB, or decrease anti-TB agent-associated toxicity [24], [25]. At present we do not know whether ND-09759 or similar drugs can meet these criteria but our studies do indicate that the combination of ND-09759 with the first-line drugs INH or RMP produced a relatively rapid reduction in mycobacterial burden in the lung and spleens after a 4-week chemotherapy. However the sterilizing activity of ND-09759 is unknown as is its ability to target non-replicating bacteria. Non-replicating *M. tuberculosis* are characterized by phenotypic and metabolic changes leading to an antibiotic-tolerant subpopulation of bacilli. Unfortunately, the process that gives rise to this population of bacteria is still unclear but likely involves environmental changes within granulomas such as hypoxia and/or exposure to reactive oxygen species [26]–[30]. The standard multi-drug chemotherapy can kill drug-susceptible, replicating *M. tuberculosis* but has limited efficacy against non-replicating *M. tuberculosis*. Consequently, the latter will likely serve as an *M. tuberculosis* reservoir contributing to the relapse of active TB following completion of therapy. Unfortunately, there is not a well-established mouse model to look at these important aspects of drug development. Moreover, *M. tuberculosis* infection in mice induces different pathological responses such as loosely organized granulomas and lack of caseous necrosis, which possibly could interfere with the assessment of compounds [31]–[33]. Guinea pig and non-human primate models, as well as some *in vitro* models, have been used to address the question of sterilizing activity and the ability to target non-replicating *M. tuberculosis*. ND-09759 or other imidazo[1,2-*a*]pyridines will need to be evaluated using these model systems. We will also continue our current efforts to develop compounds with a greater therapeutic window (>10x compared to 2x) but still effective *in vivo* at a low dose (<50 mg/kg).

Previous studies have implicated the ubiquinol cytochrome C reductase (*qcrB*) as the target of the imidazo[1,2-*a*]pyridines [9]. This was determined through genomic sequencing of spontaneous BCG resistant mutants and identification of a common target, *qcrB*, as well as the over-expression of QcrB in BCG resulting in a >10 fold increase in MIC to different imidazo[1,2-*a*]pyridines compounds. Pethe *et al*. also found their IPA Q203 targets the qcrB [10]. At present it is unclear whether there are additional targets for imidazo[1,2-*a*]pyridines but the ability of this family of compounds to target a major component of the electron transport chain shows great promise as a mechanism of action.

In summary, our studies demonstrate that ND-09759, an elaborated imidazo[1,2-*a*]pyridine carboxyamide, has bactericidal activity *in vivo* against an established *M. tuberculosis* infection at a similar dose as INH and RMP using an aerosol mouse infection model. Future studies will focus on using combination regimens containing ND-09759 or other IPAs and first-line or second-line anti-TB drugs to evaluate their activity against various drug-susceptible and drug–resistant *M. tuberculosis* clinical isolates using a suitable animal model. Given their tolerability and activity in mice, efforts should continue in the clinical evaluation of imidazo[1,2-*a*]pyridines to assess their potential to treat TB.
